# Suppression of cell migration by phospholipase C-related catalytically inactive protein-dependent modulation of PI3K signalling

**DOI:** 10.1038/s41598-017-05908-7

**Published:** 2017-07-14

**Authors:** Satoshi Asano, Yuri Taniguchi, Yosuke Yamawaki, Jing Gao, Kae Harada, Hiroshi Takeuchi, Masato Hirata, Takashi Kanematsu

**Affiliations:** 1 Department of Cellular and Molecular Pharmacology, Division of Basic Life Sciences, Institute of Biomedical and Health Sciences, Hiroshima University, 1-2-3 Kasumi, Minami-ku, Hiroshima, 734-8553 Japan; 2 Laboratory of Molecular and Cellular Biochemistry, Faculty of Dental Science, Kyushu University, Fukuoka, 812-8582 Japan; 3 Division of Applied Pharmacology, Kyushu Dental University, Kitakyushu, 803-8580 Japan; 4 Fukuoka Dental College, Fukuoka, 814-0193 Japan

## Abstract

The metabolic processes of phosphatidylinositol 4,5-bisphosphate [PI(4,5)P_2_] into PI(3,4,5)P_3_ and the subsequent PI(3,4,5)P_3_ signalling are involved in cell migration. Dysfunctions in the control of this pathway can cause human cancer cell migration and metastatic growth. Here we investigated whether phospholipase C-related catalytically inactive protein (PRIP), a PI(4,5)P_2_-binding protein, regulates cancer cell migration. PRIP overexpression in MCF-7 and BT-549 human breast cancer cells inhibited cell migration *in vitro* and metastasis development *in vivo*. Overexpression of the PRIP pleckstrin homology domain, a PI(4,5)P_2_ binding motif, in MCF-7 cells caused significant suppression of cell migration. Consistent with these results, in comparison with wild-type cells, *Prip*-deficient mouse embryonic fibroblasts exhibited increased cell migration, and this was significantly attenuated upon transfection with a siRNA targeting p110α, a catalytic subunit of class I phosphoinositide 3-kinases (PI3Ks). PI(3,4,5)P_3_ production was decreased in *Prip*-overexpressing MCF-7 and BT-549 cells. PI3K binding to PI(4,5)P_2_ was significantly inhibited by recombinant PRIP *in vitro*, and thus the activity of PI3K was downregulated. Collectively, PRIP regulates the production of PI(3,4,5)P_3_ from PI(4,5)P_2_ by PI3K, and the suppressor activity of PRIP in PI(4,5)P_2_ metabolism regulates the tumour migration, suggesting PRIP as a promising target for protection against metastatic progression.

## Introduction

The cellular phosphoinositide metabolism pathway and its metabolite signalling are critically important for numerous cellular processes. Phosphatidylinositol 4,5-bisphosphate [PI(4,5)P_2_] constitutes about only 1% of total plasma membrane lipids, but is an important lipid as a membrane-bound anchoring molecule and the precursor of three second messengers: inositol 1,4,5-trisphosphate [Ins(1,4,5)P_3_], diacylglycerol and phosphatidylinositol 3,4,5-trisphosphate [PI(3,4,5)P_3_]. These phospholipids and the metabolites are major signalling messengers of basic cellular processes, such as cell differentiation, proliferation, mitosis, migration, and cell survival. In addition, several studies have shown that dysfunctions in the control of PI(4,5)P_2_/PI(3,4,5)P_3_ signalling can often cause cancer^1^.

PI(4,5)P_2_ is converted to PI(3,4,5)P_3_ by the class I subclass of phosphatidylinositol 3-kinases (PI3Ks) that are activated by extracellular stimuli, including platelet-derived growth factor (PDGF) and epidermal growth factor. Gain-of-function mutations in PI3K [i.e., mutations in the p110 catalytic subunit of PI3K (PIK3CA)] thus enhance the PI(3,4,5)P_3_ signalling pathway and are frequently found in breast cancers and other cancers^2, 3^. The phosphatase and tensin homologue (PTEN) phosphatase enzyme dephosphorylates PI(3,4,5)P_3_ and terminates PI3K-PI(3,4,5)P_3_-mediated signalling. Accordingly, loss-of-function mutations in the PTEN gene are also frequent in human cancers^1^. These activated PI(3,4,5)P_3_ pathways induce cancer cell growth and motility, resulting in enhanced cancer cell migration and invasion^1, 4^.

Orchestrated cell motility is oriented by polarised intracellular signalling, which forms protrusive structures, such as lamellipodia and filopodia, at the leading edge of cells^5^. This process is regulated by PI(3,4,5)P_3_ signalling in controlling the actin cytoskeleton in mammalian cells. PI3K-mediated production of PI(3,4,5)P_3_ promotes the translocation of WASP family verprolin homologues protein 2 (WAVE2) to the plasma membrane and regulates guanine nucleotide exchange factor Rac-mediated actin filament remodelling^6^. WAVE2, a nucleation-promoting factor, modulates actin filament nucleation by bringing together actin monomers and pre-existing actin filaments at sites of active cytoskeletal assembly. PI(3,4,5)P_3_ directly binds WAVE2, which mediates membrane phosphoinositide signalling^7^. WAVE2 drives lamellipodium formation by enhancing actin nucleation via the actin-related protein 2 and 3 (ARP2/3) complex^8^. Higher levels and coexpression of WAVE2 and ARP2 were found in human metastatic lung adenocarcinoma and breast carcinoma, and were closely associated with poorer patient outcome^9, 10^.

Phospholipase C (PLC)-related catalytically inactive protein (PRIP) was originally isolated as a cytosol protein that binds with Ins(1,4,5)P_3_ through its pleckstrin homology (PH) domain^11, 12^. PRIP also binds to PI(4,5)P_2_ via its PH domain and localises to the plasma membrane^13–15^. We previously reported that Ins(1,4,5)P_3_ is stabilised from enzymatic metabolism by binding with PRIP in the cytosol, and thus PRIP regulates Ins(1,4,5)P_3_-Ca^2+^ signalling by Ins(1,4,5)P_3_ receptors^13, 16^. PRIP exhibits high homology to PLCδ1, but lacks PLC activity^12, 17, 18^. There are two isoforms of PRIP in mammals: PRIP1 is expressed mainly in the brain and lung, and PRIP2 is ubiquitously expressed^19, 20^. Several studies have demonstrated that PRIP regulates the intracellular trafficking machinery and fat metabolism^21–27^. However, the functions of PRIP in the regulation of phosphoinositide signalling have not been determined.

The PI(4,5)P_2_-binding but enzymatically inactive protein PRIP may regulate PI(4,5)P_2_/PI(3,4,5)P_3_ signalling. Thus, in this study, we examined the potential role and mechanisms of PRIP in controlling phosphoinositide metabolism and migration activity of cancer cells.

## Results

### PRIP1 inhibits the motility of MCF-7 and BT-549 breast cancer cells

**Figure 1:**
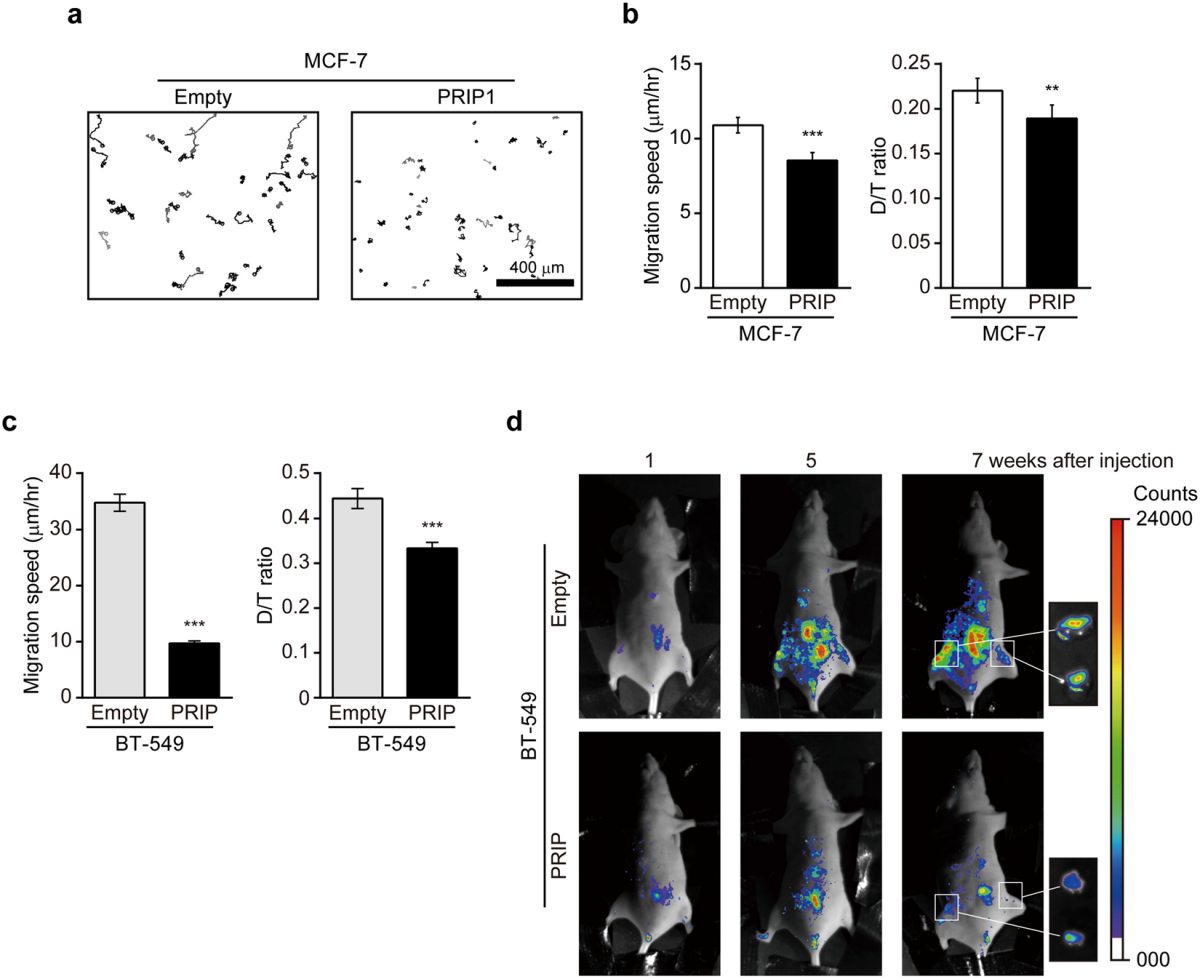
Effect of PRIP expression on cancer cell migration and metastasis. (**a**) Motility of MCF-7 cells transiently expressing either empty vector (empty) or EGFP-tagged *Prip1* (PRIP1) was monitored by microscopy after stimulation with medium containing 10% FBS. Representative track plots of cells are shown. Similar data were obtained from three independent experiments. (**b**,**c**) Random migration assays were performed using MCF-7 cells (**b**) and BT-549 cells (**c**) that stably expressed EGFP-tagged *Prip1* or DsRed2-tagged *Prip1*, respectively. Cell migration was monitored by microscopy after stimulation with 10% FBS-containing medium. Cells expressing the corresponding empty vector were used as controls. Bar graphs show comparisons of migration speed and D/T ratio (see Supplementary Fig. S2a). Chemokinesis assays were performed at least three times for each indicated experiment. Data are presented as means ± SEM. The number of samples in empty and PRIP analyses is n = 73 and 122 (**b**), and n = 100 and 241 (**c**), respectively. **p < 0.01, ***p < 0.001 versus empty vector (Mann–Whitney *U* test). (**d**) Female BALB/c-nu mice (n = 3 per group) were injected with BT-549 cells stably expressing either the empty vector or DsRed2-tagged *Prip1* (5 × 10^6^ cells/injection) into subcutaneous tissue in the vicinity of the inguinal mammary fat pad. The panels show fluorescent images of DsRed2-expressing BT-549 cells *in vivo* at weeks 1, 5, and 7. The small panels on the far right show images of lymph nodes isolated from the mice at 7 weeks post-injection (areas within the squares).

To first investigate whether PRIP expression affects cancer cell motility, we examined the cell migration of MCF-7 cells, a human breast cancer cell line. MCF-7 cells contain a heterozygous E545K mutation in exon 9 of p110α of PI3K, which abrogates the interaction between p85 and p110 subunits, resulting in constitutive activation of the PI3K pathway in MCF-7 cells^28–30^. *PRIP2*, but not *PRIP1*, was detected in MCF-7 cells by reverse transcriptase-PCR analysis (Supplementary Fig. S1a). Cell migration assays showed that MCF-7 cells transiently transfected with EGFP-*Prip1* moved shorter distances than cells transfected with the empty EGFP vector (Fig. 1a). We next generated MCF-7 cells stably expressing EGFP-*Prip1* and performed chemokinesis assays. Consistent with the results shown in Fig. 1a, the migration speed and D/T ratio (an index of the directness of cell trajectories; see Supplementary Fig. S2a) were lower in EGFP-*Prip1*-expressing MCF-7 cells than in EGFP vector-expressing cells (Fig. 1b).

The breast cancer cell line BT-549 lacks PTEN, a PI(3,4,5)P_3_ phosphatase, and thus shows excessive PI(3,4,5)P_3_ accumulation at the plasma membrane^31^. BT-549 cells expressed both *PRIP1* and *PRIP2* mRNAs (Supplementary Fig. S1b). To examine the effect of PRIP expression on cell migration of BT-549 cells, we generated BT-549 cells stably overexpressing DsRed2-*Prip1* or DsRed2-empty vector. Chemokinesis assay results showed that PRIP1 expression inhibited the migration speed by approximately one-third and reduced the D/T ratio compared to control cells (Fig. 1c). Together these data suggest that PRIP may regulate PI3K-PI(3,4,5)P_3_-induced cancer cell migration.

### PRIP inhibits the metastatic ability of BT-549 cells *in vivo*

Malignant tumours that are aggressive can invade and damage surrounding tissues. The spread of cancer cells frequently includes the invasion of local lymph nodes through the lymphatic system, and aggressive tumour cells typically enter the bloodstream and reach distant tissues^32^. To confirm the importance of PRIP expression in breast cancer metastasis *in vivo*, BT-549 cells stably expressing DsRed2-*Prip1* or vector were injected into the mammary fat pad of BALBc nude mice. At 7 weeks after the injection, empty vector-transfected BT-549 cells spread and were localised to the regional lymph nodes (Fig. 1d). However, mice injected with DsRed2-*Prip1*-expressing BT-549 cells displayed little lymphatic metastasis at 7 weeks, suggesting that PRIP1 expression suppresses the metastasis of BT-549 cells.

### PRIP is involved in PDGF-induced cell migration via PI3K

**Figure 2:**
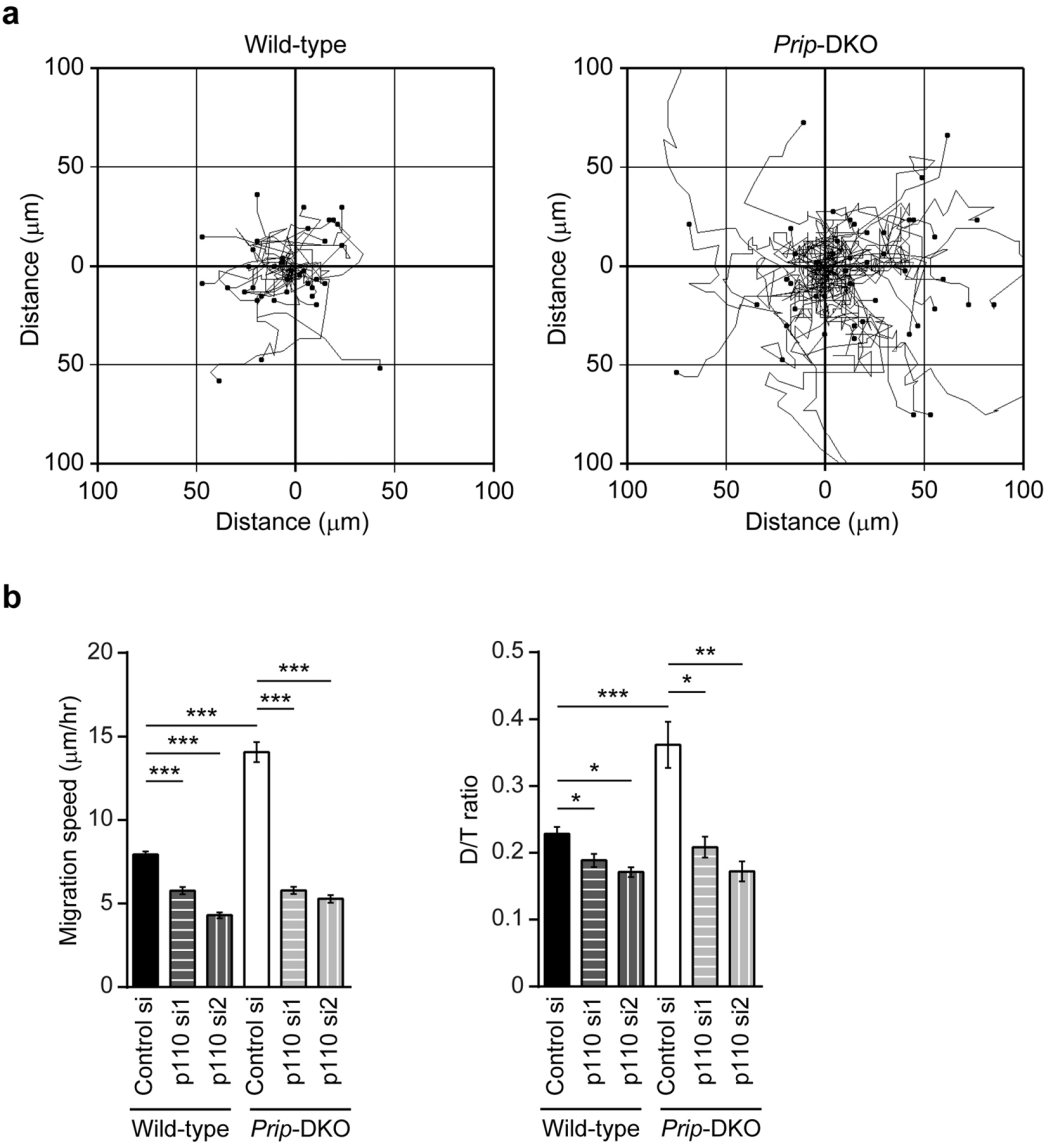
PRIP participates in PI3K-mediated cell migration. (**a**,**b**) Chemokinesis assay. Random migration of MEFs was monitored by microscopy after stimulation with 20 ng/mL PDGF (**a**). Representative track plots from at least three independent experiments are shown. Mouse *Pik3ca*-siRNAs (p110 si1 and p110 si2) and control siRNA (control si) were used for the experiments (**b**). Bar graphs show comparisons of migration speed and D/T ratio. Chemokinesis assays were performed at least three times for each indicated experiment. Data are presented as means ± SEM. The number of samples of control si, p110 si1, and p110 si2 is n = 25, 25, and 25 in wild-type, and n = 25, 23, and 21 in *Prip*-DKO, respectively. *p < 0.05, **p < 0.01, ***p < 0.001 between the indicated bars (Kruskal–Wallis test followed by Dunn’s multiple comparison test).

To investigate PRIP roles in cell movement, we performed chemokinesis and chemotaxis assays in wild-type and *Prip1* and *Prip2* double-knockout (*Prip*-DKO) mouse embryonic fibroblasts (MEFs) using PDGF-BB, a strong cell migration inducer. Chemokinesis assays showed that *Prip*-DKO MEFs displayed increased random migration activity compared with wild-type MEFs (Fig. 2a). In the chemotaxis assay, cell mobility of *Prip*-DKO MEFs was increased and the mean forward migration index in *Prip*-DKO MEFs was about 2-fold higher than that of wild-type MEFs (Supplementary Fig. S2b,c). These data suggested that PRIP deficiency enhances cell mobility and directionality.

Cell migration is regulated by PI3K signalling^2^. We thus next examined whether PRIP is involved in PI3K-PI(3,4,5)P_3_-induced cell migration by performing chemokinesis assays in MEFs inactivated for PI3K signalling. We transfected wild-type and *Prip*-DKO MEFs with synthetic siRNAs targeting p110α, the class I PI3K catalytic subunit (mouse *Pik3ca*-siRNA; p110α si1 and si2). Western blot analysis confirmed that p110α protein expression was efficiently reduced to around 30% in the siRNA-transfected MEFs compared with the MEFs transfected with control siRNA (Supplementary Fig. S3a). AKT (also known as protein kinase B) is an immediate downstream mediator of PI3K. Western blot analysis confirmed inhibition of Thr308 phosphorylation of AKT in *Pik3ca*-siRNA-transfected cells treated with PDGF stimulation, suggesting that PI3K signalling was successfully downregulated by *Pik3ca*-siRNA transfection.

In the PDGF-induced chemokinesis assays performed in control siRNA-transfected MEFs, the average migration speed and D/T ratio were 2-fold higher and 1.5-fold greater in *Prip*-DKO MEFs than in wild-type MEFs, respectively (control si, Fig. 2b). *Pik3ca* siRNA transfection inhibited the PDGF-induced changes in the migration speed and D/T ratio in both wild-type and *Prip*-DKO MEFs. Importantly, the reduced levels in *Prip*-DKO MEFs were similar to those of wild-type MEFs (Fig. 2b). Together these data suggest that PRIP is involved in PDGF-induced PI3K-mediated cell migration.

### Cell migration is suppressed by the expression of PH domain-containing PRIP1

**Figure 3:**
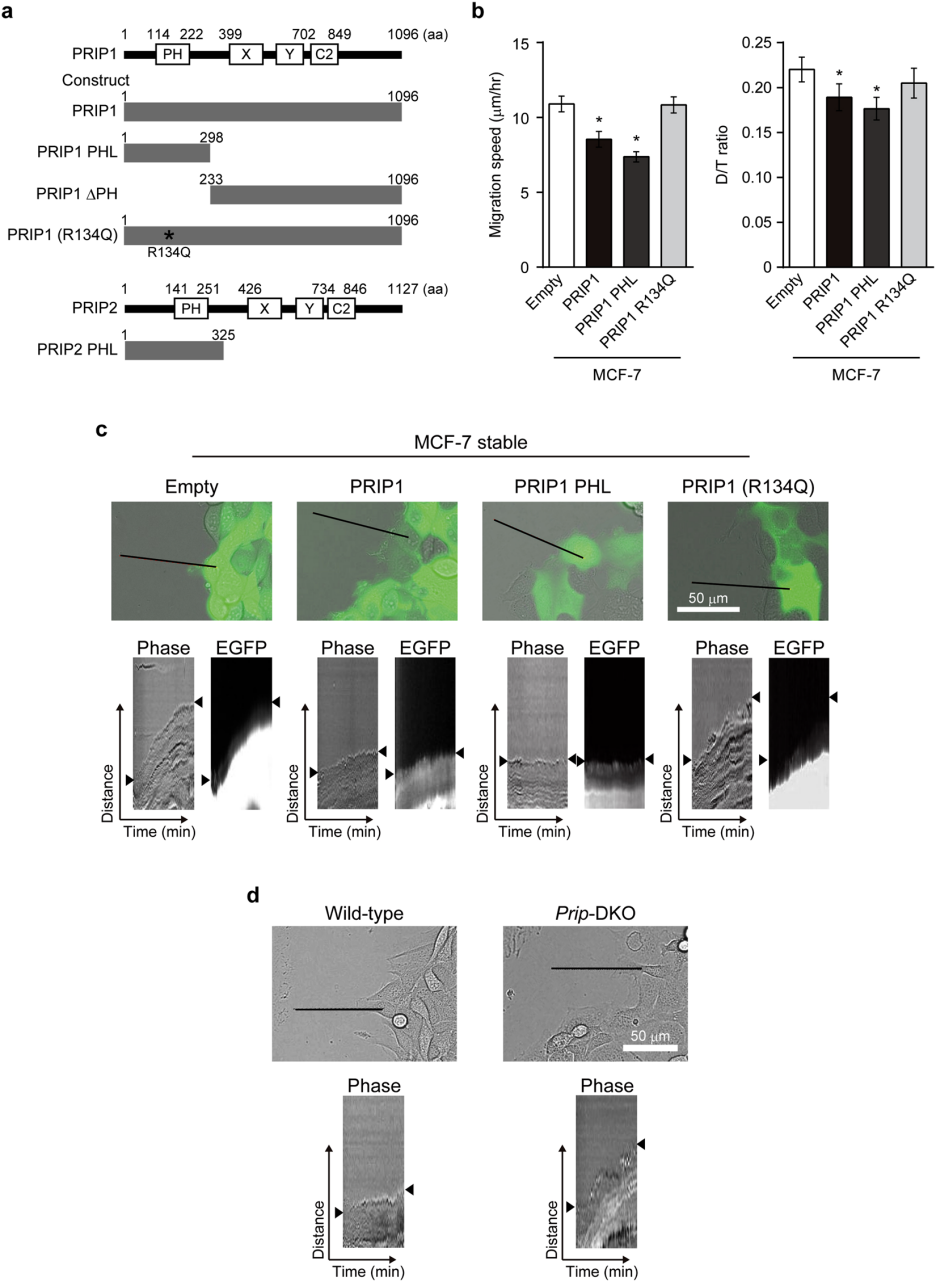
Pleckstrin homology domain of PRIP participates in the downregulation of cell migration and lamellipodium extension. (**a**) A schematic diagram of the constructs of PRIP1 mutants (upper panels) and PRIP2 mutant (lower panels). PRIP consists of a pleckstrin homology (PH) domain, X and Y domains, and C2 domain. The numbers indicate the number of amino acid (aa) residues. (**b**) The migration speeds or D/T ratios of MCF-7 cells transfected with the indicated EGFP-tagged PRIP mutants are shown. The data were obtained from three independent experiments, and are presented as means ± SEM (n = 73, 140, 104, and 79 in the left-to-right direction on each graph). *p < 0.05 versus empty vector (Kruskal–Wallis test followed by Dunn’s multiple comparison test). (**c**,**d**) PRIP regulates lamellipodium extension at the leading edge of migrating cells. The extension of the leading edge (arrowheads in the lower panels) on MCF-7 cells **(c)**, which stably expressed either the empty vector or indicated EGFP-tagged constructs, and wild-type and *Prip*-DKO MEFs (**d**) was monitored during wound healing. The images were acquired at 1-min intervals for 60 min. The kymographs (lower panels) were analysed at the black lines in the upper panels. Similar results were obtained from three independent experiments and representative images are shown.

To identify the functional domain of PRIP involved in the regulation of cell migration, EGFP-tagged *Prip1* or EGFP-tagged *Prip1* truncation mutants (Fig. 3a) were transfected into MCF-7 cells and migration assays were performed. Transfection of full-length *Prip1* or the *Prip1* PHL N-terminal truncation mutant, which contains the PH domain, resulted in a significantly reduced migration speed and D/T ratio compared with MCF-7 cells transfected with the EGFP-empty vector. Importantly, the PRIP1 R134Q mutant, which does not bind PI(4,5)P_2_^33^, failed to inhibit migration speed and D/T ratio compared with the empty control (Fig. 3b).

We then performed a scratch wound closure assay to investigate the effect of PRIP1 on lamellipodium extension at the leading edge of migrating cells and analysed lamella dynamics at the leading edge by kymography. Dynamic lamellipodia were extended in MCF-7 cells expressing EGFP alone or the PRIP1 R134Q mutant, but were obviously inhibited in cells expressing full-length PRIP1 or PRIP1 PHL (Fig. 3c). Importantly, EGFP signals in cells expressing PRIP1 and PRIP1 PHL, but not PRIP1 R134Q, were detected at the edge of migrating cancer cells, suggesting PRIP1 containing the PH domain functions at the leading edge.

**Figure 4:**
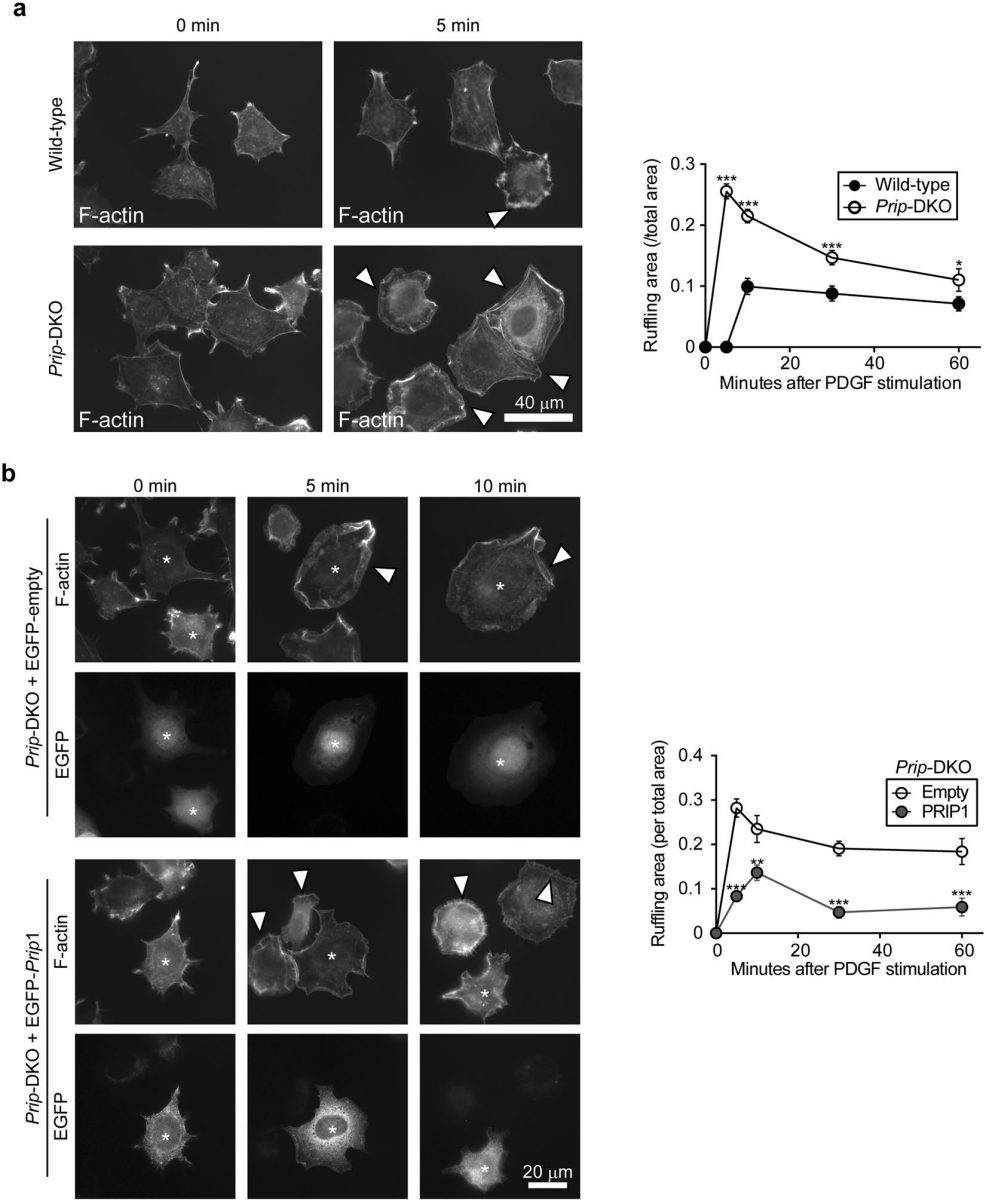
PRIP regulates PDGF-induced cytoskeletal remodelling. (**a**,**b**) Wild-type and *Prip*-DKO MEFs (**a**) and *Prip*-DKO MEFs transfected with EGFP vector (empty) or EGFP-*Prip1* (**b**) were grown on a fibronectin-coated dish and starved for 3 h prior to stimulation with 20 ng/mL PDGF for the indicated period of time. F-actin was stained with Alexa Fluor 350 phalloidin. Arrowheads indicate ruffling membrane (**a**,**b**). Asterisks indicate EGFP-expressing cells (**b**). Similar data were obtained from at least three independent experiments, and representative images are shown. The graphs on the right show the membrane ruffling area relative to the total cell area. The data are presented as means ± SEM determined at 0, 5, 10, 30, and 60 min [(**a**) wild-type, n = 27, 27, 20, 18, and 23; *Prip-*DKO, n = 27, 13, 23, 13, and 14; (**b**) empty, n = 22, 6, 12, 12, and 10; EGFP-*Prip1*, n = 27, 10, 11, 11, and 14, respectively, in the left-to-right direction on the graph]. *p < 0.05, **p < 0.01, ***p < 0.001 versus the corresponding wild-type (**a**) and empty vector (**b**) values (Mann–Whitney *U* test).

PDGF stimulation-induced lamellipodia formation also extended further in *Prip*-DKO MEFs than in wild-type MEFs in a scratch wound closure assay (Fig. 3d). Transfection of *Prip1* PHL or *PRIP2* PHL into *Prip*-DKO MEFs decreased the migration speed and D/T ratio compared with cells transfected with EGFP empty vector (Supplementary Fig. S4a). However, transfection of *Prip1* ΔPH (PRIP1 lacking the N-terminal and PH domain) or *Prip1* R134Q failed to decrease migration speed or D/T ratio. Activated PI3K induces membrane ruffling^34^; therefore, a cell spreading assay was performed to examine the involvement of PRIP in PI3K signalling. The area of membrane ruffling in PDGF-induced cell extension was increased in *Prip*-DKO MEFs compared with wild-type MEFs during 60 min after stimulation (Fig. 4a). The enhanced membrane ruffles in *Prip*-DKO MEFs were attenuated by transfection with *Prip1* (Fig. 4b).

To examine the localisation of PRIP, *Prip*-DKO MEFs were transfected with various EGFP-tagged *Prip1* mutants. Full-length PRIP1 as well as PRIP1 PHL and PRIP2 PHL accumulated at the plasma membrane in transfected *Prip*-DKO MEFs after PDGF stimulation (5 min) (Supplementary Fig. S4b). In contrast, the EGFP signals for PRIP1 ΔPH and PRIP1 R134Q were distributed in the cytosol, and the cells transfected with these plasmids displayed lamellipodia formation. Taken together, these results suggest that PRIP localises in the plasma membrane via the PH domain, affects PI3K activation-mediated actin remodelling, and inhibits the formation of lamellipodia.

### PRIP regulates the biosynthesis of PI(3,4,5)P_3_ during lamellipodia formation

**Figure 5:**
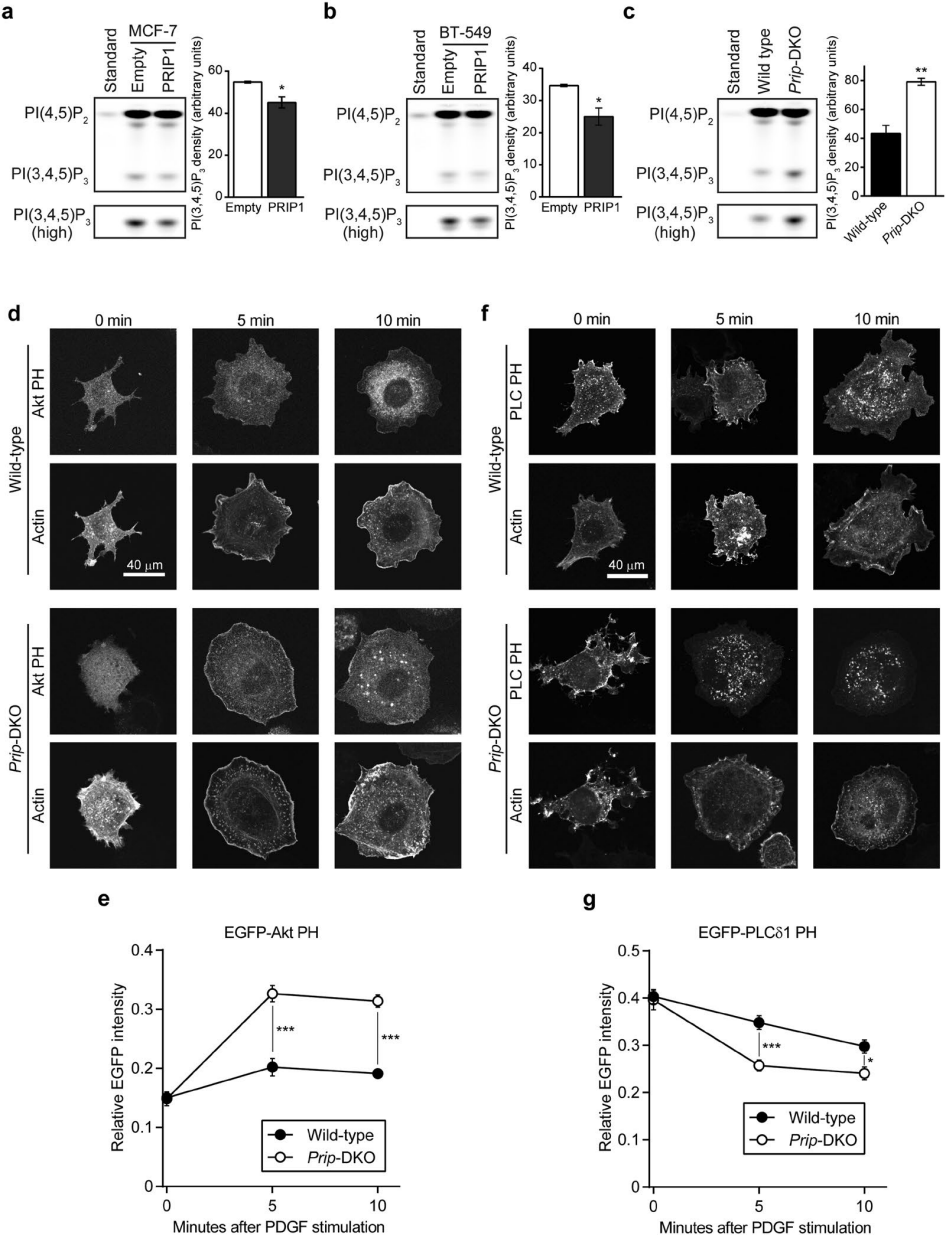
PRIP regulates the alteration of PI(3,4,5)P_3_ and PI(4,5)P_2_ on the plasma membrane. **(a–c)** MCF-7 (**a**) and BT-549 (**b**) cells stably expressing EGFP-tagged *Prip1* and DsRed2-tagged *Prip1*, respectively, and cells transfected with empty vectors (**a**,**b**) and wild-type and *Prip*-DKO MEFs **(c)** were used for experiments. Cells were incubated with BODIPY FL-PI(4,5)P_2_ for 1 h in medium containing 10% FBS (**a**,**b**) or 20 ng/mL PDGF (**c**). The lipids extracted from cells were distinguished by thin layer chromatography. The first lane in each panel (standard) shows the chromatographic mobility of 1 nmol fluorescent PI(4,5)P_2_. The lower panels were obtained using a high sensitivity mode (high). Similar data were obtained from three independent experiments, and representative images are shown. The PI(3,4,5)P_3_ bands were quantified using ImageJ software. Data are presented as means ± SEM [**(a**–**c**), n = 3 per group]. (**d**–**g**) MEFs transfected with either EGFP-tagged Akt PH (**d**,**e**) or EGFP-tagged PLCδ PH (**f**,**g**) and Kusabira-Orange-tagged actin were stimulated with 20 ng/mL PDGF for the indicated period of time. Similar data were obtained from at least three independent experiments, and representative images are shown (**d**,**f**). Fluorescence intensity of EGFP-Akt PH **(e)** and EGFP-PLCδ PH (**g**) on the plasma membrane was measured and normalised by the intensity of whole cells. The data are presented as means ± SEM determined at 0, 5, and 10 min [(**e**) wild-type, n = 36, 23, and 36; *Prip*-DKO, n = 38, 58, and 58; (**g**) wild-type, n = 41, 34, and 20; and *Prip*-DKO, n = 26, 49, and 19, respectively, in the left-to-right direction on the graph]. *p < 0.05, **p < 0.01, ***p < 0.001 versus the corresponding control value (**a**–**c**) or between the indicated bars (**e**,**g**) (Mann–Whitney *U* test).

To investigate the involvement of PRIP in PI(3,4,5)P_3_ biosynthesis, we analysed phospholipid metabolism in MCF-7 and BT-549 cells using a subcellular PI3K activity assay. The metabolism of PI(4,5)P_2_ to PI(3,4,5)P_3_ was inhibited in MCF-7 and BT-549 cells upon overexpression of PRIP1 by approximately 75% or 65% compared with controls, respectively (Fig. 5a,b). Conversely, more PI(3,4,5)P_3_ was detected in *Prip*-DKO MEFs compared with that in wild-type MEFs after stimulation with PDGF (Fig. 5c).

We next examined changes in PI(3,4,5)P_3_ and PI(4,5)P_2_ on the plasma membrane after PDGF stimulation using wild-type and *Prip*-DKO MEFs that overexpressed an EGFP-tagged Akt PH domain (EGFP-Akt PH), a probe for PI(3,4,5)P_3_, and an EGFP-tagged PLCδ1 PH domain (EGFP-PLCδ1 PH), a probe for PI(4,5)P_2_^35^. Accumulation of EGFP-AKT PH signals on the plasma membrane of both wild-type and *Prip*-DKO MEFs reached a plateau at 5 min after PDGF stimulation (Fig. 5d,e). Greater accumulation of EGFP-AKT PH was detected in *Prip*-DKO MEFs than in wild-type MEFs at 5 min and 10 min post-treatment with PDGF. PDGF treatment of both wild-type and *Prip*-DKO MEFs caused a decrease of EGFP-PLCδ1 PH on the plasma membrane, with a greater reduction observed in *Prip*-DKO MEFs compared with wild-type MEFs (Fig. 5f,g). Together these data suggest that PRIP is involved in regulating the production of PI(3,4,5)P_3_.

### Acceleration of PI(3,4,5)P_3_-mediated downstream signalling in *Prip*-DKO MEFs

**Figure 6:**
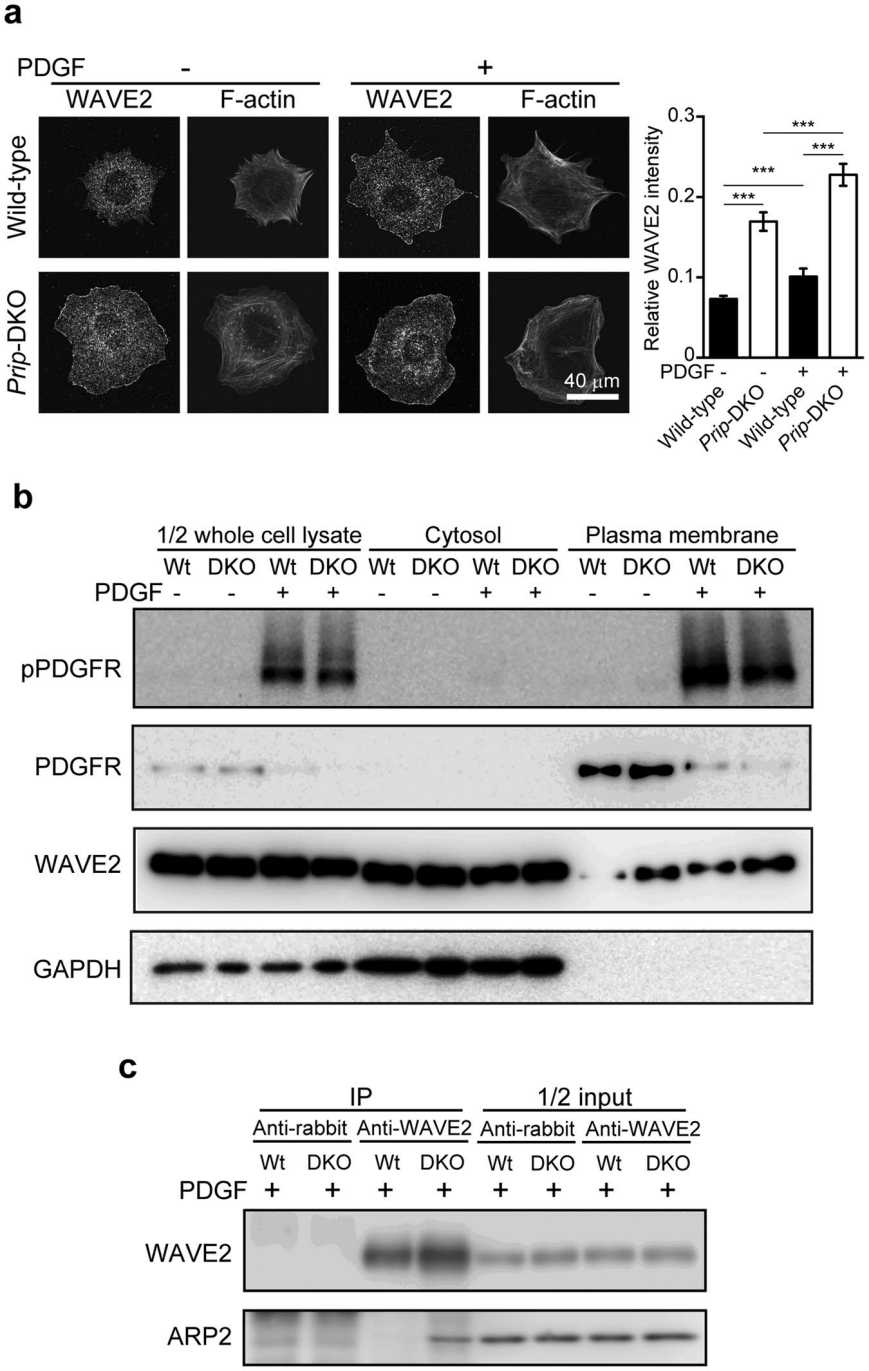
Alteration of PI(3,4,5)P_3_-mediated downstream signalling. (**a**–**c**) MEFs were stimulated with (+) or without (−) 20 ng/mL PDGF for 10 min. Cells were evaluated by immunocytochemistry using an anti-WAVE2 antibody and Alexa Fluor 350 phalloidin (**a**), cell fractionation assay by centrifugation (**b**), and immunoprecipitation (IP) assay using an anti-WAVE2 antibody (**c**). All experiments were performed at least three times independently, and similar data were obtained. Representative images are shown (**a**–**c**). The graph in (**a**) shows the fluorescence intensity of WAVE2 on the plasma membrane normalised by the intensity for the whole cell. The data are presented as means ± SEM [n = 39, 24, 34, and 24 in the left-to-right direction on the graph (**a**)]. ***p < 0.001 between the indicated bars (Kruskal–Wallis test followed by Dunn’s multiple comparison test). The detection of GAPDH and PDGFR in (**b**) represents a quality assessment of the purification.

WAVE2 binds to PI(3,4,5)P_3_ on the plasma membrane followed by interactions with the ARP2/3 complex, which promotes actin polymerisation^7^. To investigate whether the upregulation of PI(3,4,5)P_3_ in *Prip*-DKO MEFs affects WAVE2 localisation to the plasma membrane, we performed immunocytochemistry with an anti-WAVE2 antibody and phalloidin staining to visualise F-actin (Fig. 6a). WAVE2 signals on the plasma membrane were stronger in *Prip*-DKO MEFs than wild-type MEFs without PDGF stimulation. For both genotypes, PDGF stimulation promoted the localisation of WAVE2 to the plasma membrane; however, the *Prip*-DKO MEFs displayed more apparent translocation of WAVE2 (right graph, Fig. 6a).

To further verify WAVE2 translocation to plasma membrane fractions, we performed centrifugal fractionation analyses (Fig. 6b). Glyceraldehyde 3-phosphate dehydrogenase (GAPDH) and PDGF receptor (PDGFR) were specifically detected in the cytosol fractions and the plasma membrane fractions, respectively, confirming the success of the fractionation. The expression of PDGFR was similar between wild-type and *Prip*-DKO MEFs (see whole cell lysate in Fig. 6b). In both wild-type and *Prip*-DKO MEFs, PDGFR was detected in plasma membrane fractions and was reduced by PDGF stimulation, suggesting that PDGFR is endocytosed by PDGF stimulation^36^. Since the phosphorylation levels of PDGFR in response to PDGF stimulation were similar in both cell lines, the PDGF-mediated PDGFR activation mechanism appears to be intact in both genotypes. Consistent with the results of the immunocytochemical assay, in basal conditions, greater accumulation of WAVE2 was observed in the plasma membrane fractions of *Prip*-DKO MEFs than wild-type MEFs (Fig. 6b). In response to PDGF stimulation, WAVE2 displayed further additional accumulation in the membrane fraction of *Prip*-DKO MEFs compared with that of wild-type MEFs.

We then examined the interaction between WAVE2 and ARP2 using an immunoprecipitation assay with an anti-WAVE2 antibody. In response to PDGF stimulation, a larger amount of ARP2 coprecipitated with WAVE2 in *Prip*-DKO MEFs than in wild-type MEFs (Fig. 6c).

### PRIP expression attenuates the PI(3,4,5)P_3_ signalling pathway

The PI3K-PI(3,4,5)P_3_-AKT pathway can be activated after growth factor stimulation. Activated PI3K recruits AKT to the plasma membrane by binding between the AKT PH domain and PI(3,4,5)P_3_, which induces AKT Thr308 phosphorylation, followed by AKT Ser473 phosphorylation by mTORC2^37^. In MCF-7 cells, the phosphorylation levels of AKT at Thr308 and Ser473 were reduced in cells expressing EGFP*-*PRIP1 compared with control cells (Supplementary Fig. S3b). *Prip*-DKO MEFs displayed enhanced AKT phosphorylation with PDGF stimulation (Supplementary Fig. S3a). These data suggested that the PI3K-PI(3,4,5)P_3_-AKT signalling pathway is downregulated in PRIP1-overexpressing cells and upregulated in *Prip*-deficient cells. AKT activation leads to cell proliferation^38^. Indeed, PRIP deficiency in MEFs upregulated cell proliferation in culture medium supplemented with 2% or 5% foetal bovine serum (FBS), whereas *Prip1*-expressing MCF-7 cells exhibited inhibited cell proliferation in medium with 2% or 5% FBS (Supplementary Fig. S5a,b).

### PRIP suppresses the conversion of PI(4,5)P_2_ to PI(3,4,5)P_3_ by PI3K

**Figure 7:**
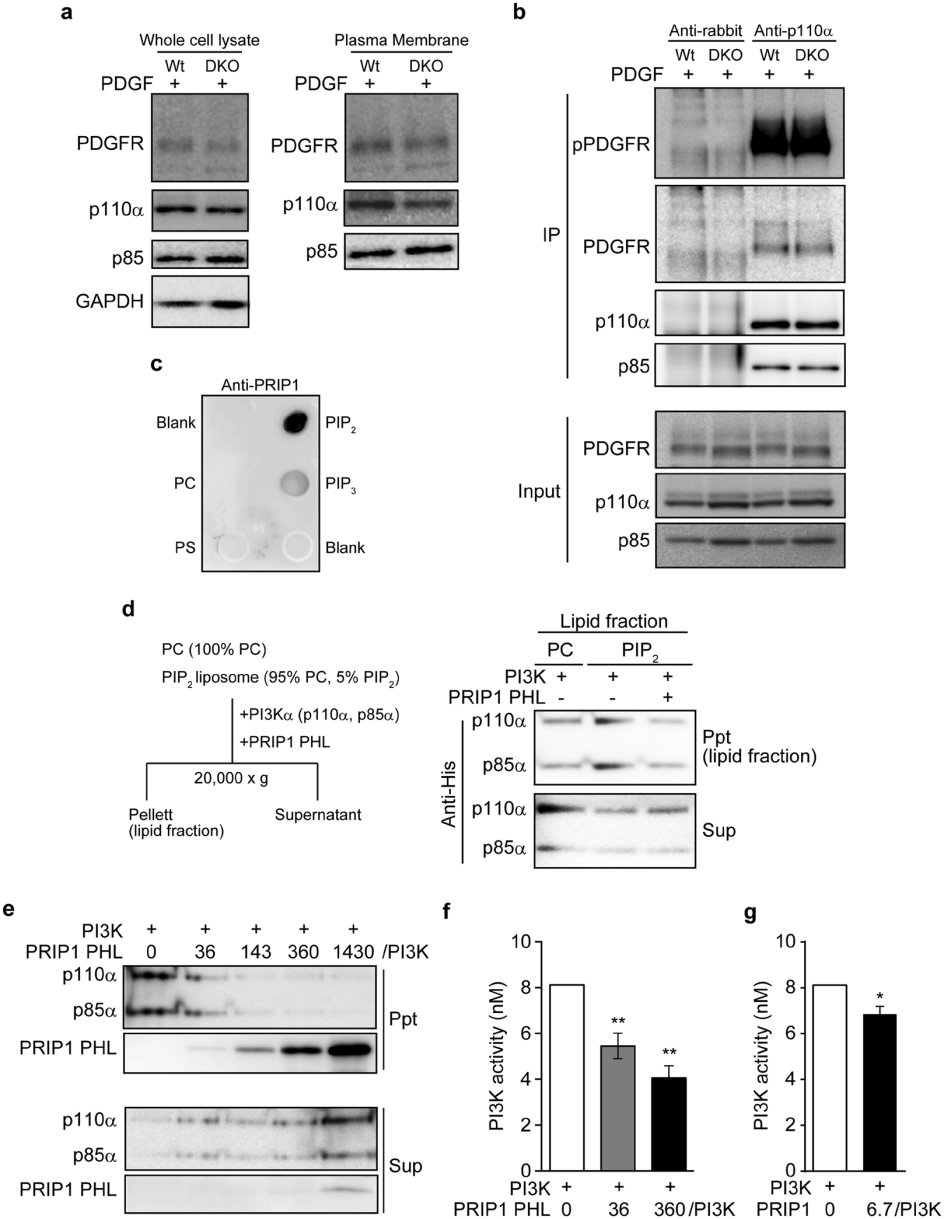
PRIP suppresses the conversion of PI(4,5)P_2_ to PI(3,4,5)P_3_ by PI3K. (**a**,**b**) MEFs were stimulated (+) with 20 ng/mL PDGF for 10 min. Treated cells were homogenised and fractionated by centrifugation (**a**) or lysed and immunoprecipitated (IP) with an anti-p110α antibody (**b**). Western blotting was performed with the indicated specific antibodies. Similar data were obtained from at least three independent experiments, and a set of representative images is shown. (**c**) Specific binding of EGFP-PRIP1 to PI(4,5)P_2_. Indicated lipids were blotted on a polyvinylidene difluoride membrane. Signals were detected using anti-PRIP1 antibody. PIP_2_, PI(4,5)P_2_; PIP_3_, PI(4,5)P_3_; PC, phosphatidylcholine; PS, phosphatidylserine. (**d**,**e**) A PI(4,5)P_2_ sedimentation assay was performed using recombinant His-tagged PI3Kα in the presence (+) or absence (−) of recombinant PRIP1 PHL (see Supplementary Fig. S6a) (**d**), or in the presence of the indicated dose of the PRIP1 PHL (**e**). The method is schematically presented on the left in (**d**). Liposomes are composed of PI(4,5)P_2_: PC = 5:95 (molar ratio) (PIP_2_) or 100% PC (PC). The obtained lipid fractions (ppt) and supernatants (sup) were evaluated by western blotting using an anti-His antibody (for PI3Kα) and anti-GST antibody (for PRIP1 PHL). Similar data were obtained from three independent experiments, and representative images are shown. **(f**,**g)** Metabolic activity of PI3Kα in the production of PI(4,5)P_3_ in the presence of PRIP1 PHL (**f**) or recombinant PRIP1 (Supplementary Fig. S6b) (**g**). The data are presented as means ± SEM [(**f**,**g**) n = 3 for each indicated experiment]. *p < 0.05, **p < 0.01 versus the control without PRIP1 PHL (**f**) or PRIP1 (**g**) [Kruskal–Wallis test followed by Dunn’s multiple comparison test (**f**) or Mann–Whitney *U* test (**g**)].

To examine the effects of PRIP on the distribution of PI3Kα (p110α and p85 subunits), we performed a cell fractionation assay by centrifugation, followed by western blotting (Fig. 7a). Similar amounts of p110α and p85 subunits of PI3K were detected in the plasma membrane fractions after PDGF stimulation for 10 min in both wild-type and *Prip*-DKO MEFs. PDGF activates PDGFR on the cell membrane and induces complex formation between PDGFR and PI3K via the Src homology 2 domains of the p85 regulatory subunit, which binds to phosphorylated tyrosine on the activated PDGFR^39^. To investigate whether PRIP is involved in forming the complex between PDGFR and PI3K, we performed an immunoprecipitation assay with anti-p110α using MEF cell lysates (Fig. 7b). Similar amounts of PDGFR and PI3K p85 were detected in p110α immunoprecipitates, with similar PDGFR phosphorylation levels, in both genotypes. These data suggest that PDGF-induced PDGFR activation followed by complex formation between PDGFR and PI3K is not regulated by PRIP.

PRIP preferentially bound PI(4,5)P_2_, but not PI(3,4,5)P_3_ or other lipids, *i.e*., phosphatidylcholine (PC) and phosphatidylserine (Fig. 7c). We next examined if PRIP affects the interaction between PI3K and PI(4,5)P_2_. An *in vitro* co-sedimentation assay was performed using liposomes composed of 100% PC or 5% PI(4,5)P_2_ and 95% PC (Fig. 7d). Both His-tagged p110α and p85α (PI3K complex) were precipitated with the PI(4,5)P_2_-PC liposomes (Fig. 7d), and the association was dose-dependently inhibited by PRIP1 PHL (Fig. 7e), suggesting that PRIP interferes with the substrate recognition of PI3K. We then investigated whether PRIP1 PHL affects PI3K substrate metabolism. ELISA assays in evaluating PI3K activity towards PI(3,4,5)P_3_ showed reduced PI3K activity in response to PRIP1 PHL in a dose-dependent manner (Fig. 7f) and in response to PRIP1 (Fig. 7g), indicating that PRIP negatively regulates the PI3K-PI(3,4,5)P_3_ production pathway.

## Discussion

Metastasis remains the major driver of mortality in patients with cancer. A comprehensive understanding of the molecular processes governing cell migration and invasion is necessary for the development of new anti-metastatic therapies. In this study, we revealed a novel role of PRIP in cancer cell migration as a co-modulator of PI(4,5)P_2_ in the plasma membrane. The introduction or acceleration of PRIP gene/protein expression may promise applications for the reduction of patient morbidity and mortality.

The constitutive activation of the PI3K/AKT signalling cascade, which typically occurs by mutations in genes encoding receptor tyrosine kinases *PI3K*, *PTEN*, or *AKT*, is very common in cancer. Deregulation of PI3K activity (e.g., H1047R and E542K in p110α) is associated with high malignancy rates and an increased resistance to chemotherapeutic and radiation therapies against breast cancer^2, 4^. The reduced expression or ablation of PTEN, a PI(3,4,5)P_3_ phosphatase, also results in malignant transformation^2, 4^. The breast cancer cell lines MCF-7 and BT-549 have a mutation in exon 9 of *PIK3CA* and a loss of *PTEN*, respectively, and display a PI(3,4,5)P_3_ overproduction-linked malignant phenotype^3, 31^. Therefore, we used these two cell lines to investigate the involvement of PRIP in PI(3,4,5)P_3_ signalling. Our results demonstrate that the introduction of *Prip1* into MCF-7 and BT-549 cells inhibits cancer cell migration and membrane extension, and this action was required for PRIP binding to PI(4,5)P_2_ via the PH domain (Figs 1 and 3). Furthermore, the ablation of *Prip* from MEFs upregulated cell migration and membrane extension (Figs 2, 3, 4, and Supplementary Fig. S4), suggesting inhibitory effects of PRIP on cell migration.

PRIP1 was originally purified from rat brain cytosol^11^ and has since been isolated from membrane fractions^14^. A previous study showed that recombinant PRIP1 PH domain binds to Ins(1,4,5)P_3_ with *K*_*D*_ = 15.6 μM and glycerophosphoinositol 4,5-diphosphate with *K*_*D*_ = 125 μM *in vitro* ^40^. In this study, EGFP-tagged PRIP1 preferentially bound to PI(4,5)P_2_ rather than PI(3,4,5)P_3_ (Fig. 7c), and PRIP1 and PRIP1 PH localised to the leading edge of the plasma membrane during lamellipodia-based migration (Fig. 3c), suggesting that PRIP localises to PI(4,5)P_2_ and regulates PI(3,4,5)P_3_ production. Consistently, PI(3,4,5)P_3_ accumulation and higher production-mediated downstream signalling were observed in *Prip*-deficient cells compared with normal cells (Figs 5 and 6). In addition, the PI(4,5)P_2_ metabolism via PI3K was inhibited by the PRIP PH domain (Fig. 7). These data indicated that PRIP prevents the PI3K-mediated conversion of PI(4,5)P_2_ into PI(3,4,5)P_3_.

The overexpression of *Prip1* in MCF-7 and BT-549 cells disrupted both the speed and directionality of cell migration (Fig. 1b,c, and PRIP1 lanes in Fig. 3b). These effects were mimicked by the introduction of the PRIP-PH domain mutant (Fig. 3b). Consistent with these results, *Prip*-deficient MEFs displayed accelerated cell migration and directionality compared with wild-type MEFs (control siRNA lanes in Fig. 2b). These enhanced effects were inhibited by the introduction of PRIP1 and PRIP2 PHL domain mutants, but not inhibited by the PRIP1 ΔPH mutant or the PRIP1 R134Q mutant, which does not bind PI(4,5)P_2_ (Supplementary Fig. S4). These data indicate that cell migration activity is primarily regulated by the PRIP PH domain via binding with PI(4,5)P_2_. Furthermore, overexpression analysis using PLCδ PH showed differences in phospholipid metabolism between *Prip*-DKO and wild-type MEFs, suggesting that PLCδ PH cannot rescue the *Prip*-DKO phenotype to the normal phenotype (Fig. 5f,g). In contrast, PRIP PHL rescued PRIP functions in *Prip*-DKO MEFs, indicating that phosphoinositide signalling via PRIP is specific for the PHL domain of PRIP. Interestingly, PRIP1 PHS, a short PH domain mutant, showed different localisation compared with the PRIP1 PHL in MEFs (Supplementary Fig. S4b,c). PRIP1 PHS signals displayed weak localisation on the plasma membrane; however, lamellipodia formation was inhibited after PDGF stimulation. Therefore, the PRIP N-terminal region (1–74 aa) is needed to express the specific function of PRIP PH in phospholipid metabolism.

Migration is a polarised cellular process, and the polarity can regulate cell mobility in a specific direction^41^. Our scratch wound closure assays revealed that the expression of PRIP inhibited protrusive front edge formation, which was regulated by actin remodelling via PI3K-WAVE2-ARP2/3 signalling (Figs 3 and 6). Moreover, cell migration is regulated not only by WAVE2-ARP2/3 signalling, but also by phosphorylated AKT (activated form), which promotes myosin II assembly followed by actomyosin contractions by mediating p21-activated kinase phosphorylation^42, 43^. Thus, a dramatic modulation of PI(3,4,5)P_3_ production coordinated by PRIP1 and PRIP2 may also regulate AKT-mediated cell motility. Further investigations are needed.

Epithelial-mesenchymal transition (EMT) is a developmental process in which epithelial cells acquire migratory and invasive properties^44^. Mesenchymal markers are expressed in BT-549 cells but not in MCF-7 cells^45^. Our result showed that stably overexpressed PRIP in BT-549 cells suppressed the metastatic ability (Fig. 1d). Importantly, the overexpression partially reversed the expression pattern of EMT markers (Supplementary Fig. S7), suggesting PRIP is a negative regulator for EMT in breast cancer cells. However, PRIP roles in EMT program need further investigation.

**Figure 8:**
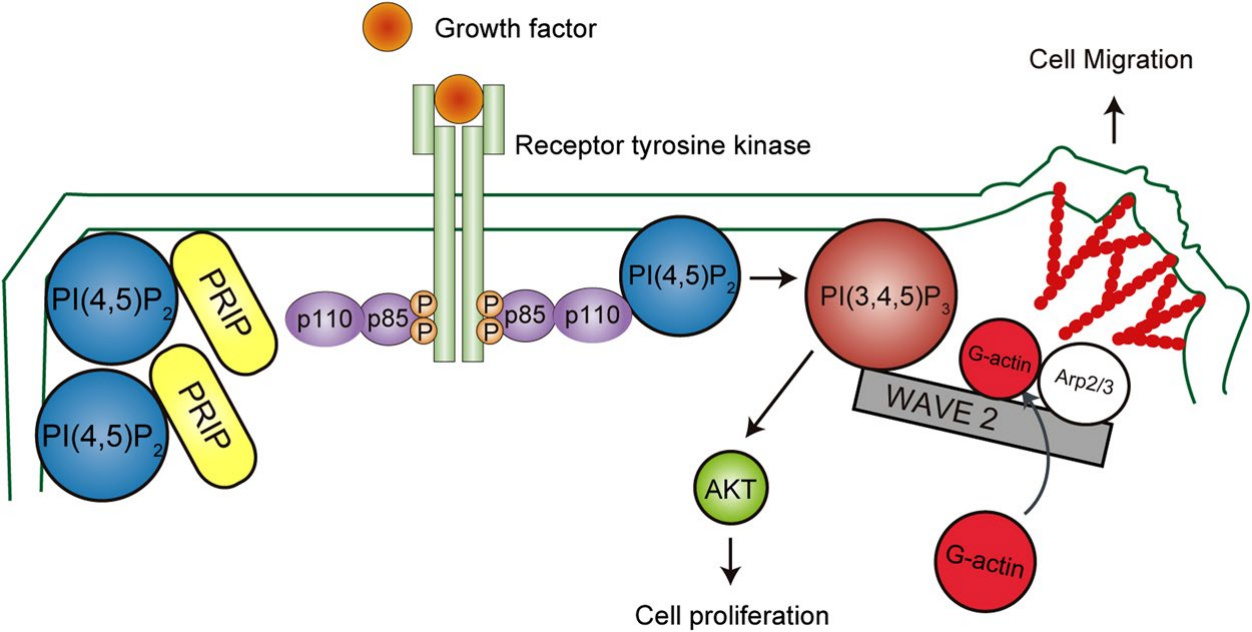
Schematic representation of PRIP-mediated cell migration. PRIP binds to PI(4,5)P_2_ via its PH domain and sequesters PI(4,5)P_2_ from a metabolizing enzyme PI3K. This inhibits the interaction between p110α, catalytic subunit of PI3K, and PI(4,5)P_2_. When PI3K is activated by growth factor stimulation, the excessive metabolism to PI(3,4,5)P_3_ is protected by the proper access of the PI3K to PI(4,5)P_2_. PRIP is a negative modulator for PI(3,4,5)P_3_-WAVE2-induced actin polymerization and cell migration. PI3K: a heterodimer between a p110 catalytic subunit and a p85 regulatory subunit.

This is the first study to examine the roles of PRIP in phospholipid metabolism and cell migration. Our findings conclusively demonstrated that PRIP can regulate cancer cell migration activity *in vitro* and metastasis development *in vivo*. The novel tumour migration suppressor activity of PRIP was preferentially correlated with PI3K-PI(3,4,5)P_3_ production signalling. As a key regulatory mechanism, we propose that the association of PRIP with PI(4,5)P_2_ suppresses the conversion of PI(4,5)P_2_ into PI(3,4,5)P_3_ by PI3K (Fig. 8). Hence, this new molecule regulating phosphoinositide metabolism is a potential therapeutic target for protection against metastatic progression.

## Methods

### Plasmids and siRNAs

EGFP-tagged PLCδ1 PH, Akt PH, *Prip1* (full-length rat *Prip1* gene), *Prip1* PHL, *Prip1* ΔPH, *Prip1* R134Q and *PRIP2* PHL (human *PRIP2* PH domain gene), DsRed-*Prip1* (full-length), and GST-tagged *Prip1* PHL were described previously^15, 46^. Kusabira-Orange-tagged β-actin (KO1-actin) was purchased from Medical & Biological Laboratories (Nagoya, Japan). Human p110α (*PIK3CA*)-siRNA (si1, 1116744; si2, 1116747) and mouse *Pik3ca*-siRNA (si1, 1408655; si2, 1408665) were purchased from Bioneer Corporation (Daejeon, Korea). A negative control siRNA (S10C-0600) was purchased from Cosmo Bio (Tokyo, Japan).

### Antibodies

Anti-ARP2 (#5614), anti-GAPDH (#2118), anti-PDGF receptor β (#3169), anti-phospho-PDGF receptor β (Tyr751; #4549), anti-PI3 kinase p85 (#4257), anti-PI3-kinase p110α (#4249), anti-WAVE2 (#3659), and HRP-conjugated anti-rabbit IgG (#7074) were purchased from Cell Signaling Technology (Beverly, MA, USA). Anti-PRIP1 polyclonal antibody was developed previously^21^. Anti-β-actin (IMG-5142A) was obtained from Imgenex (San Diego, CA, USA). Anti-His (D291-3S), anti-GST (M209-3), and normal rabbit IgG (PM035) were obtained from Medical & Biological Laboratories. HRP-conjugated anti-mouse IgG (NA9310) was obtained from GE Healthcare (Little Chalfont, UK). Alexa Fluor 488 anti-rabbit IgG (A-11008) was obtained from Invitrogen (Carlsbad, CA, USA).

### Cell culture and transfection

The BT-549 cell line was purchased from the American Type Culture Collection (ATCC; Rockville, MD, USA). Preparation of wild-type and *Prip*-DKO MEFs was described previously^25^. MEFs were immortalised by transfecting the simian virus 40 large T antigen. Cells were cultured under conventional growth conditions and transfected with plasmids or siRNA using the Lonza 4D-Nucleofector X Unit (Lonza, Basel, Switzerland) or Lipofectamine 3000 (Invitrogen).

### Production of stable PRIP-expressing cells

Cells were transfected with the expression vectors coding EGFP-*Prip1* or DsRed2-*Prip1* and cultured in the presence of 1 mg/ml G418 (Nakarai Tesque, Kyoto, Japan) for 14 days. Stable colonies that expressed fluorescent protein were selected for further experiments.

### Random migration assay and cell spreading assay

MCF-7 and BT-549 cells (1.5 × 10^4^) were seeded on culture dishes or glass coverslips coated with fibronectin in serum-free medium, cultured until attached, and stimulated with 20 ng/mL PDGF-BB (for MEFs; Peprotech, Rocky Hill, NJ, USA) or 10% FBS (for MCF-7 and BT-549 cells). For the migration assay, cells were monitored every 30 min for 18 h by live-cell imaging (BZ-9000; Keyence, Osaka, Japan). Tracking analysis of cells was performed using Image-Pro premier ver.9.4 (Media Cybernetics, Rockville, MD, USA). For the cell spreading assay, cells were fixed, F-actin was stained with Alexa Fluor 350 phalloidin, and a confocal laser scanning microscope was used for observations (Fluoview FV10i; Olympus, Tokyo, Japan). The intensity for the cell edge and ruffling area was measured by ImageJ 1.45 s (National Institutes of Health, Bethesda, MD, USA). To observe the extension of the leading edge, cells were seeded on μ-dishes (Ibidi, Martinsried, Germany), cultured until confluent, wounded with a pipet tip, and stimulated with 20 ng/mL PDGF-BB (for MEFs) or 10% FBS (for MCF-7 cells). The cells were recorded every 1 min for 60 min by live-cell imaging on a BZ-9000 microscope. Kymography analysis of the leading edge was performed using the MultipleKymograph plug-in of ImageJ 1.45 s (National Institutes of Health; developed by J. Rietdorf and A. Seitz).

### Mouse metastasis study and ethics statement

BT-549 breast cancer metastasis was assayed by the modified method of Wiegmans *et al*.^47^. Briefly, five-week-old age-matched female nude mice (BALB/c-nu; Charles River Japan, Yokohama, Japan) were injected into the mammary fat pad with metastatic BT549 cells stably expressing DsRed2-*Prip1* or a control empty vector (5 × 10^6^ cells/100 μL of phosphate-buffered saline). Tumour growth and metastasis was visualised using NightOWL (Berthold Technologies, Bad Wildbad, Germany) for live animal imaging and monitored weekly. All procedures and animal work were approved by the Animal Care and Use Committee of Hiroshima University (permission number: A15-119) and were performed in accordance with the Guide for Hiroshima University Animal Experimentation Regulation.

### Immunofluorescence, immunoprecipitation, and western blotting

Immunofluorescence, immunoprecipitation, and western blotting were carried out following previously described methods^24^. F-actin was visualised by Alexa Fluor 350-labelled phalloidin (Invitrogen). Cell fractionation was performed using the Minute Plasmid Membrane Protein Isolation Kit (Invent Biotechnologies, Plymouth, MN, USA) according to the manufacturer’s protocol.

### Thin-layer chromatography

For the assessment of PI(3,4,5)P_3_ levels in cells, cells were incubated with mixture of 20 μM BODIPY FL-PtdIns(4,5)P_2_ (C-45F6a; Echelon, San Jose, CA, USA) and carrier 2 (P-9C2, Echelon) for 60 min at 37 °C and washed twice with ice-cold phosphate-buffered saline. Lipids were extracted by CHCl_3_/MeOH (v/v = 2:1) using the methods of Bligh and Dyer^48^, applied to thin-layer chromatography plates, and developed in CHCl_3_/acetone/MeOH/AcOH/water (v/v/v/v/v = 40:15:13:12:7) using the methods of Huang *et al*.^49^. The fluorescent lipids were visualised using the Molecular Imager FX^TM^ (Bio-Rad, Hercules, CA, USA).

### Measurement of PI(4,5)P_2_ and PI(3,4,5)P_3_ levels at the plasma membrane

KO1-Actin and EGFP-PLCδ1 PH or EGFP-Akt PH were co-expressed in MEFs, and cell spreading assay was performed as described above. The EGFP intensities on the plasma membrane was measured and normalised by the intensity of whole cells.

### Liposome sedimentation assay and measurement of PI3K activity

A binding assay of PI3K to liposomes containing with PI(4,5)P_2_ was carried out following previously described methods^15^, with modifications, using recombinant His-tagged PI3Kα (10 pg; Jena Bioscience, Jena, Germany) and PI(4,5)P_2_ (Cayman, Ann Arbor, MI, USA) and/or PC (Sigma-Aldrich, St. Louis, MO, USA) in the presence or absence of purified recombinant GST-tagged PRIP1 PHL. PI3K activity was measured *in vitro* using the PI3-Kinase Activity ELISA Kit (Echelon).

### Statistical analysis

GraphPad Prism was used for the statistical analyses. A non-parametric Mann–Whitney *U* test (for two groups) and Kruskal–Wallis test (for more than two groups) followed by Dunn’s multiple comparison test were used. A *p-*value of less than 0.05 was considered statistically significant.

### Availability of materials and data

All data generated or analysed during this study are included in this published article and its Supplementary Information file. Other relevant information is available from the authors on reasonable request.

### Ethics approval

All procedures and animal work were approved by the Animal Care and Use Committee of Hiroshima University (A15-119) and were performed in accordance with the Guide for Hiroshima University Animal Experimentation Regulation.

## Electronic supplementary material

Supplementary Information
