# Supplementary material for: Suppression of cell migration by phospholipase C-related catalytically inactive protein-dependent modulation of PI3K signalling
Source: Sci Rep. 2017 Jul 14;7:5408. doi: 10.1038/s41598-017-05908-7 (PMC5511194; doi:10.1038/s41598-017-05908-7)
Supplement: Supplementary file 1 — Supplementary Information [file 41598_2017_5908_MOESM1_ESM.pdf]

Supplementary information

Suppression of cell migration by phospholipase C-related catalytically inactive protein-dependent modulation of PI3K signalling

Satoshi Asano, Yuri Taniguchi, Yosuke Yamawaki, Jing Gao, Kae Harada, Hiroshi Takeuchi, Masato Hirata and Takashi Kanematsu

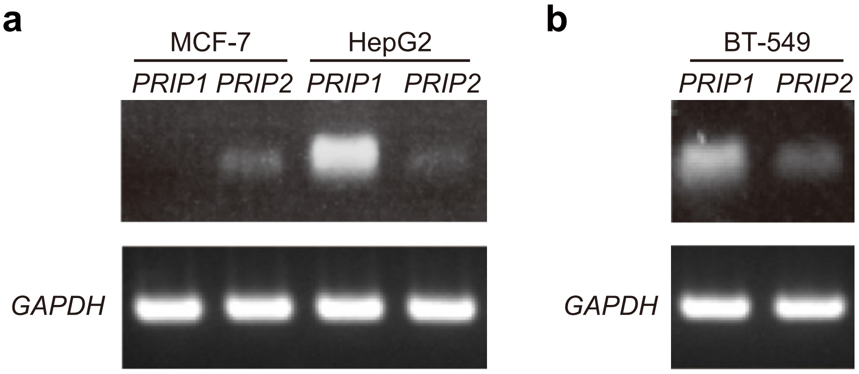

Supplementary Figure S1. Expression of *PRIP1* and *PRIP2* in MCF-7 and BT-549 cells. **(a, b)** Reverse transcriptase-PCR analysis of *PRIP1* and *PRIP2* mRNAs in MCF-7 **(a)** or BT-549 **(b)** cells. HepG2 cells were used as a positive control. *GAPDH* was used as a control. Human *PRIP1*-, *PRIP2*-, and *GAPDH*-specific sequences were amplified using the following primers: 5′-TGAGAATGGGGAAGAAAGTT-3′ and 5′-TCTATGGCTTCTCGTAATGG-3′ for *PRIP1*, 5′-ACTGTGGCTATGTCCTCCGG-3′ and 5′-TTTAATGTGAAGAAGTTGAG-3′ for *PRIP2*, and 5′-GGAGCGAGATCCCTCCAAAAT-3′ and 5′-GGCTGTTGTCATACTTCTCATGG-3′ for *GAPDH*.

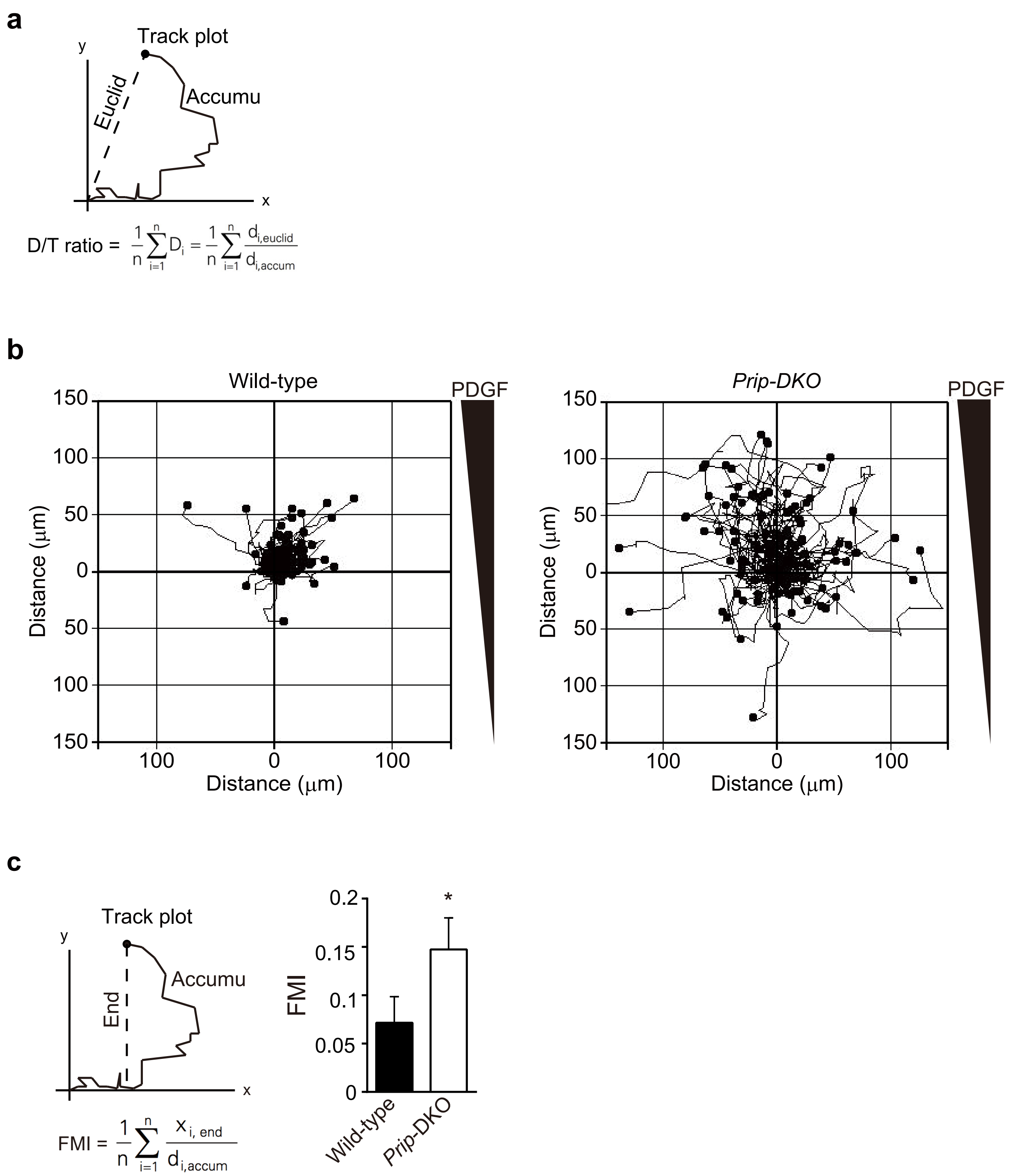

Supplementary Figure S2. Chemotaxis assays of MEFs. **(a)** Schematic representation of a track plot and the D/T ratio calculated by comparing the Euclidian distance (Euclid) to the accumulated distance (Accum) are shown. **(b)** Two-dimensional chemotaxis assay. MEF migration was measured using a μ-Slide Chemotaxis Assay (Ibidi, Martinsried, Germany) as previously described [Asano, S. *et al*. Regulation of cell migration by sphingomyelin synthases: sphingomyelin in lipid rafts decreases responsiveness to signaling by the CXCL12/CXCR4 pathway. *Mol. Cell Biol.* **32**, 3242–3252 (2012)]. Briefly, MEFs were seeded onto the μ-Slide and stimulated in a concentration gradient (from 0 to 50 ng/mL) of PDGF-BB, and then monitored by microscopy every 30 min for 18 h. Track plots of wild-type and *Prip* double-knockout MEFs (*Prip*-DKO) were analysed. The starting point for each individual cell is shown in the centre of the diagram. Similar data were obtained in three independent experiments. **(c)** Schematic representation and a formula for the forward migration index (FMI), which represents the efficiency of the forward migration of cells. FMI was compared between wild-type MEFs (n = 89) and *Prip*-DKO MEFs (n = 111) in three independent experiments. The data are presented as means ± SEM. (Unpaired t test with Welch's correction: $*p < 0.05$ versus the wild-type value).

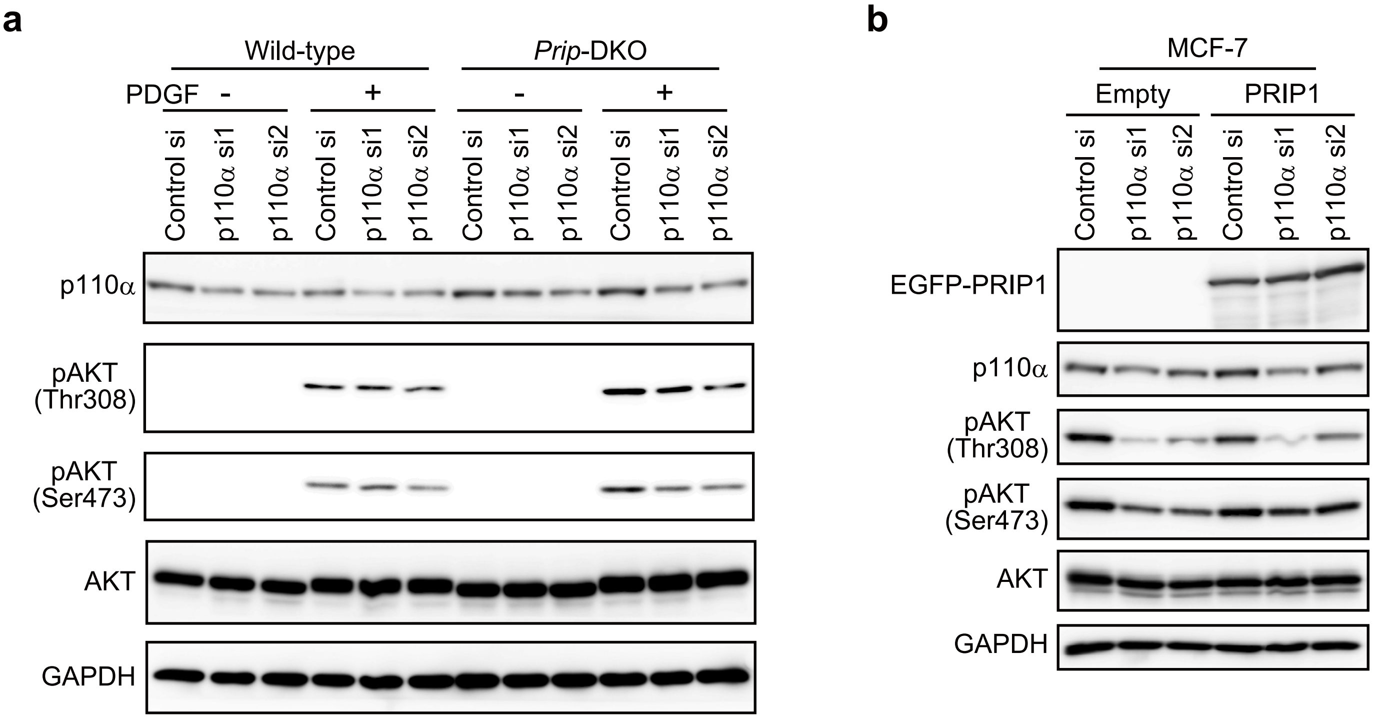

Supplementary Figure S3. Silencing of the p110α subunit of PI3K in MEFs and MCF-7 cells. **(a,b)** Cells were transfected with the indicated siRNAs: control siRNA, *Pik3ca*-siRNAs (p110α si1 and p110α si2) **(a)**, and human *PIK3CA*-siRNAs (p110α si1 and p110α si2) **(b)**. After 2 days of cell culture, the cells were treated with a culture medium with (+) or without (-) 20 ng/mL PDGF **(a)** or 10% FBS-containing culture medium **(b)** for 10 min and lysed. Whole cell lysates were analysed by western blotting with the indicated specific antibodies. Equivalent amounts of protein were loaded into each well, and GAPDH was used as a loading control. A set of typical images from three independent experiments is shown. Anti-PI3-kinase p110α (#4249), anti-Akt (pan; #4691), anti-phospho-Akt (Thr308; #2965), and anti-phospho-Akt (Ser473; #4060) were purchased from Cell Signaling Technology (Beverly, MA, USA).

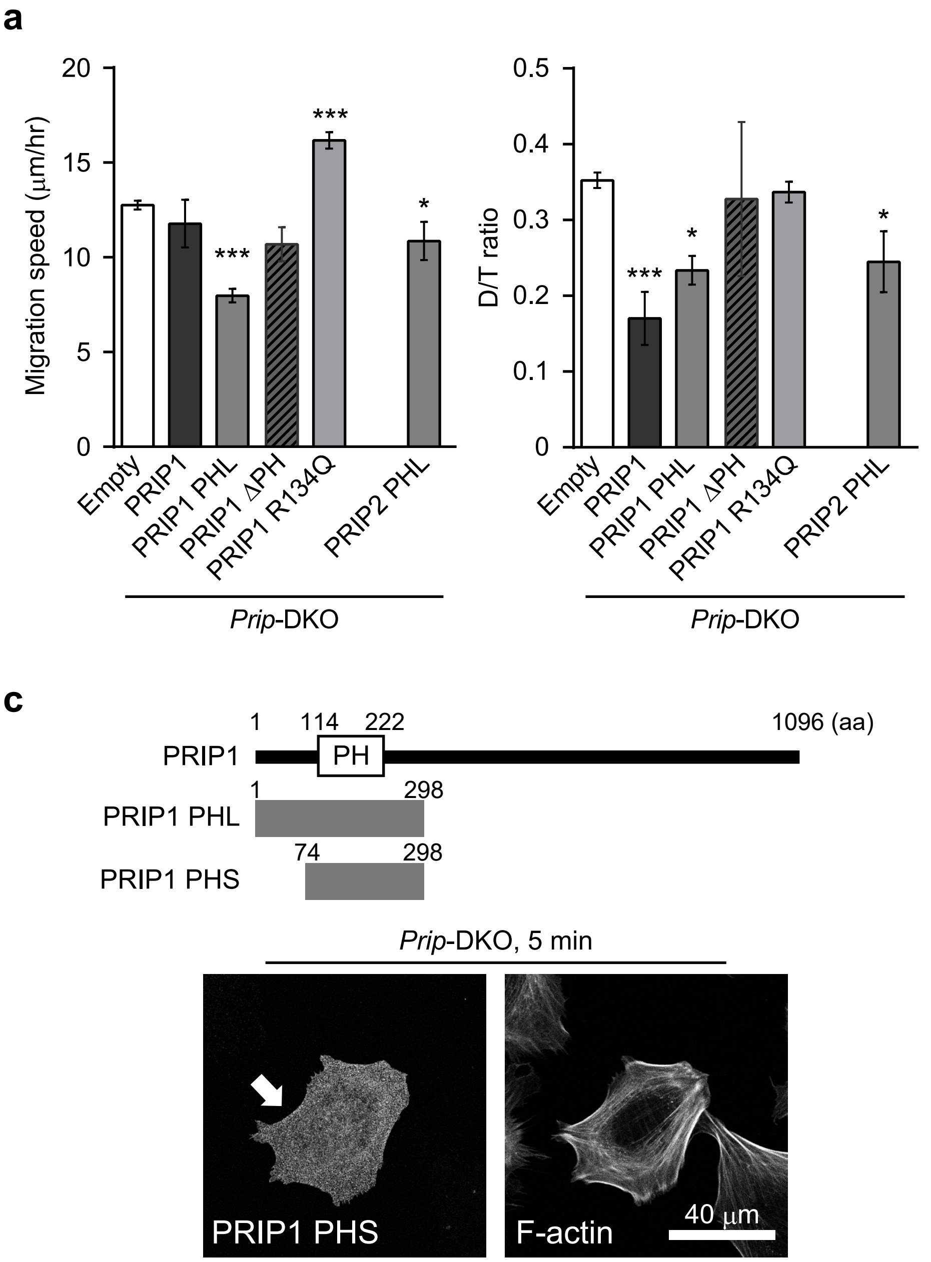

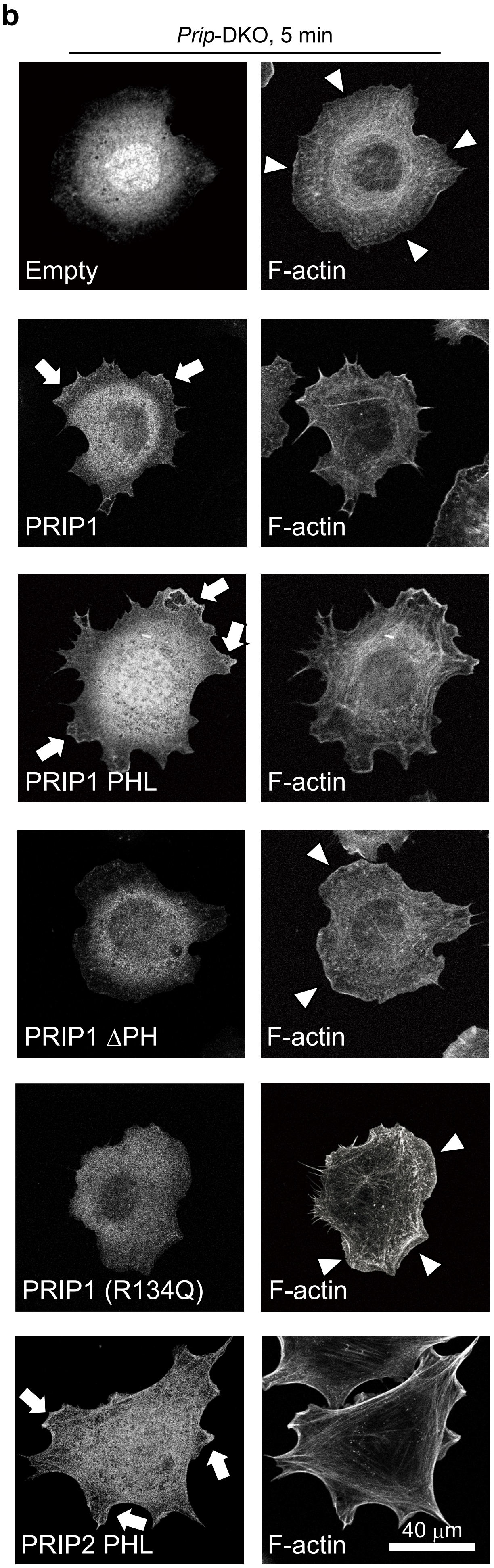

Supplementary Figure S4. The pleckstrin homology domain of PRIP is involved in the regulation of PDGF-induced MEF migration. **(a)** Random migration of MEFs was monitored by microscopy after stimulation with 20 ng/mL PDGF. The migration speed and D/T ratio of *Prip*-DKO MEFs transfected with the indicated EGFP-tagged PRIP mutants (see Fig. 3a) are shown. The data were obtained from three independent experiments, and are presented as means ± SEM (n = 77, 20, 24, 20, 71, and 23 in the left-to-right direction on the graph. *$p < 0.05$, ***$p < 0.001$ versus empty vector (Kruskal–Wallis test followed by Dunn' s multiple comparison test). **(b,c)** *Prip*-DKO MEFs transfected with the indicated EGFP-tagged PRIP mutants were stimulated with 20 ng/mL PDGF for 5 min. Localisation of each PRIP mutant and actin formation (F-actin, stained with Alexa Fluor 350 phalloidin) are represented. Arrows indicate the accumulation of EGFP signals for the PRIP mutant on the plasma membrane. Arrowheads indicate lamellipodia. Similar data were obtained from at least three independent experiments. A schematic diagram of the constructs of PRIP1 PH (**c**, upper panel). The numbers indicate the number of amino acid (aa) residues.

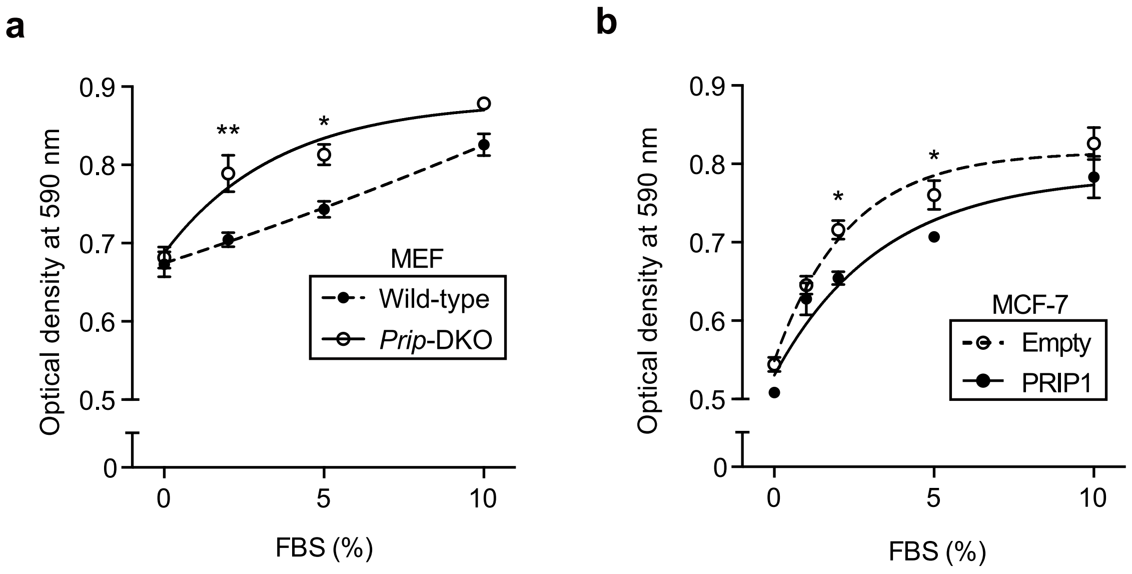

Supplementary Figure S5. Proliferation assay of MEFs and MCF-7 cells. **(a,b)** Proliferation assays for wild-type and *Prip*-DKO MEFs **(a)** and MCF-7 cells stably expressing EGFP alone or EGFP-*Prip1* (PRIP1) (b) were performed 24 h after seeding in medium with various concentrations of FBS [**(a)**, 0%, 2%, 5%, and 10%; **(b)**, 0%, 1%, 2%, 5%, and 10%] using the 3-(4,5-Di-methylthiazol-2-yl)-2,5-diphenyltetrazolium bromide (MTT) Assay Kit (Nacalai Tesque) (each experiment, n = 3).

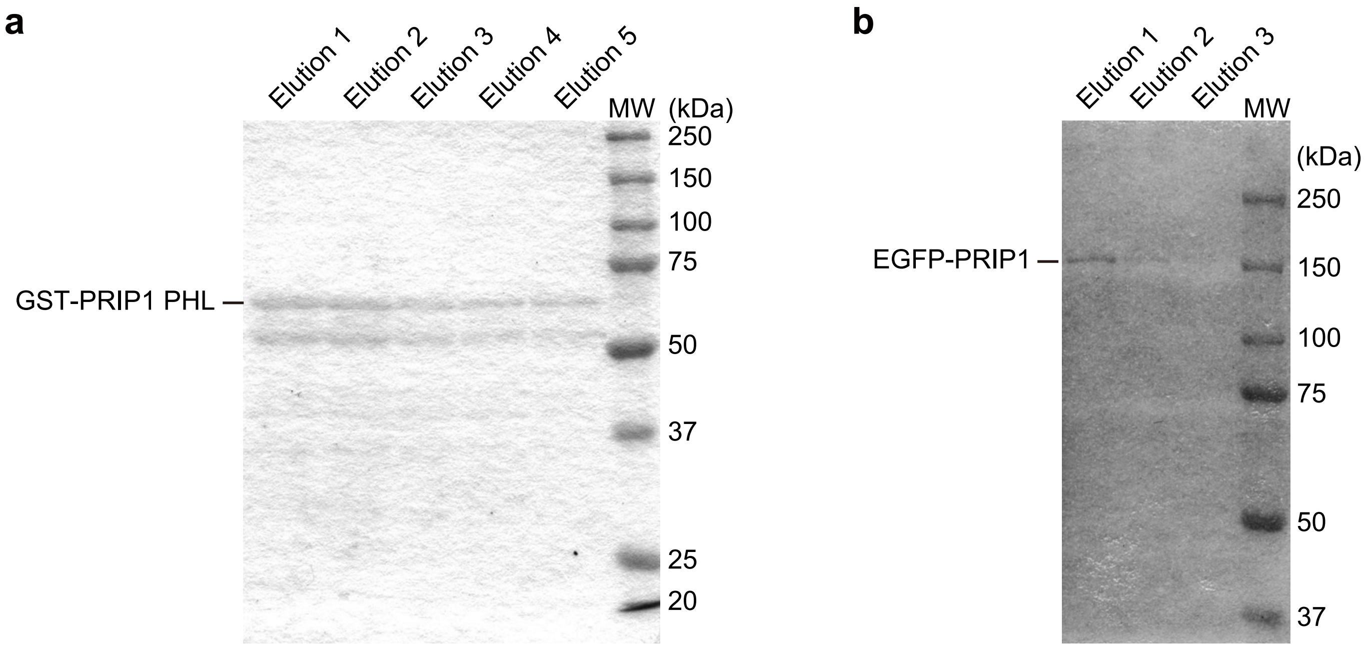

Supplementary Figure S6. Purification of PRIP1. **(a,b)** GST-tagged PRIP1 PHL was expressed in *Escherichia coli* and purified for use in PRIP-lipid binding assays (Fig. 7d–f) **(a)**. EGFP-tagged PRIP1 was purified from MCF-7 cells stably expressing EGFP-*Prip1* for use in PRIP-lipid binding assays (Fig. 7c and g) **(b)**. The sampled fractions were separated by SDS-PAGE and analysed by Coomassie Brilliant Blue staining.

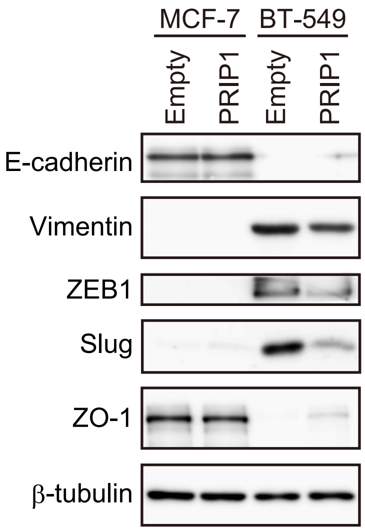

Supplementary Figure S7. PRIP is involved in epithelial-mesenchymal transition (EMT). BT-549 cells stably expressing either the empty vector or DsRed2-tagged *Prip1* were lysed. Whole cell lysates were analysed by western blotting with the indicated specific antibodies. Equivalent amounts of protein were loaded into each well, and β-tubulin was used as a loading control. MCF-7 cell line is a representative of non-invasive epithelial cancer cells. A set of typical images from three independent experiments is shown. The hallmark of EMT is the loss of E-cadherin (and ZO-1), and the acquisition of mesenchymal markers vimentin (ZEB1 and Slug). Anti-ZEB1 (#3396), anti-slug (#9585), anti-ZO-1 (#8193), anti-E-cadherin (#3195) and anti-vimentin (#5741) were purchased from Cell Signaling Technology (Beverly, MA, USA).
